# Definitive diagnosis and surgical management of pleural and diaphragmatic endometriosis: a case report

**DOI:** 10.1186/s13019-026-04266-8

**Published:** 2026-06-14

**Authors:** Andrea M. Gochi, Kenneth M. Williams, Michelle M. Kimura, Nicholas D. Sze, Jeffrey B. Velotta

**Affiliations:** 1 Department of Surgery, University of California San Francisco-East Bay, 1411 E 31 st St, Oakland, 94602 CA USA; 2 Center for Surgery and Public Health, Brigham and Women’s Hospital, MA Boston, USA; 3 John A. Burns School of Medicine, University of Hawaiʻi at Mānoa, Honolulu, HI USA; 4 Boonshoft School of Medicine, Wright State University, Fairborn, OH USA; 5 Thoracic Surgery, Kaiser Permanente Oakland Medical Center, Oakland, CA USA; 6 Department of Surgery, University of California San Francisco, San Francisco, CA USA

**Keywords:** Thoracic endometriosis, Pleural endometriosis, Diaphragmatic endometriosis, Minimally invasive surgery, Cyclical pain

## Abstract

**Background:**

Thoracic endometriosis is an uncommon and frequently under recognized manifestation of endometriosis involving the pleura, diaphragm, or lung parenchyma. Patients often present with nonspecific cyclical thoracic or upper abdominal pain, and diagnosis is frequently delayed. Surgical management with video-assisted thoracoscopic surgery (VATS) allows definitive diagnosis and surgical management of identified thoracic lesions, particularly when disease spans the thoracic and abdominal cavities.

**Case presentation:**

A 40-year-old woman with six years of cyclical right upper quadrant pain refractory to hormonal therapy was found to have suspected thoracic and diaphragmatic endometriosis on imaging. Multidisciplinary surgical management with combined VATS and laparoscopy revealed pleural, diaphragmatic, and pelvic endometrial implants, which were completely excised. Pathology confirmed thoracic and pelvic endometriosis. The patient has experienced sustained symptom resolution past 12-month follow-up.

**Conclusions:**

Thoracic endometriosis should be considered in reproductive-age women with unexplained cyclical thoracoabdominal pain. Combined minimally invasive thoracic and abdominal exploration enables comprehensive evaluation and complete resection of disease across the diaphragm, leading to durable symptom control. Early multidisciplinary recognition is essential to optimize outcomes.

## Background

Thoracic endometriosis is a rare form of endometriosis involving the pleura, diaphragm, lung parenchyma and/or airway, typically presenting with cyclical chest pain, catamenial pneumothorax, or hemothorax [1, 2]. It primarily affects women of reproductive age and results from ectopic endometrial tissue implantation within the thorax [3]. Frequently underdiagnosed due to its nonspecific symptoms [3, 4], the condition is often a diagnosis of exclusion [5].

Thoracic endometriosis demonstrates a marked right-sided predominance, reported in over 85–90% of cases [6]. This striking laterality is thought to reflect both anatomic and physiologic factors, including clockwise peritoneal fluid circulation directing endometrial cells toward the right subphrenic space, with migration through diaphragmatic fenestrations. Inspiratory negative intrathoracic pressure may also facilitate transdiaphragmatic passage of endometrial tissue into the right pleural cavity [6].

Video-assisted thoracoscopic surgery (VATS) plays a central role in both diagnosis and management, allowing direct visualization, resection of lesions, repair of diaphragmatic defects, and symptom resolution [7]. Given the high prevalence of concurrent pelvic endometriosis, reported in up to 50–80% of cases, combined thoracic and abdominal evaluation through a multidisciplinary team is often warranted [7–9].

We present a case of thoracic endometriosis involving the pleura, diaphragm, and lung parenchyma in a patient with a prolonged, atypical presentation characterized by isolated cyclical right upper quadrant (RUQ) pain, successfully managed with a single-stage, minimally invasive multidisciplinary approach.

## Case report

A 40-year-old woman with a history of perforated appendicitis treated with open appendectomy and postoperative perihepatic abscess requiring drainage and prolonged intravenous antibiotics presented with a 6-year history of cyclical RUQ pain without pulmonary manifestations. She did not have this pain prior to her appendiceal surgery. After treatment of the postoperative abscess, the pain initially resolved for a period of time, but later recurred in a cyclical rather than clearly catamenial pattern. Histopathologic results from the prior appendectomy specimen were not available for review, as that surgery occurred at an outside hospital. Multiple hormonal therapies were trialed with only partial or transient improvement. Prior non-suppressive hormonal therapy, including oral contraceptives and levonorgestrel intrauterine device placement in early 2021, reduced the severity of her worst pain episodes but did not eliminate daily pain. She was later treated with suppressive hormonal therapy using elagolix (Orilissa) twice daily beginning in mid 2023, with marked improvement during the first 6 weeks; however, pain recurred by 2 months later in 2023, and the medication was discontinued because durable relief was not achieved. Although she was not menstruating while on elagolix, she reported that pain continued to intensify cyclically around the time when menses would otherwise have been expected.

**Fig. 1:**
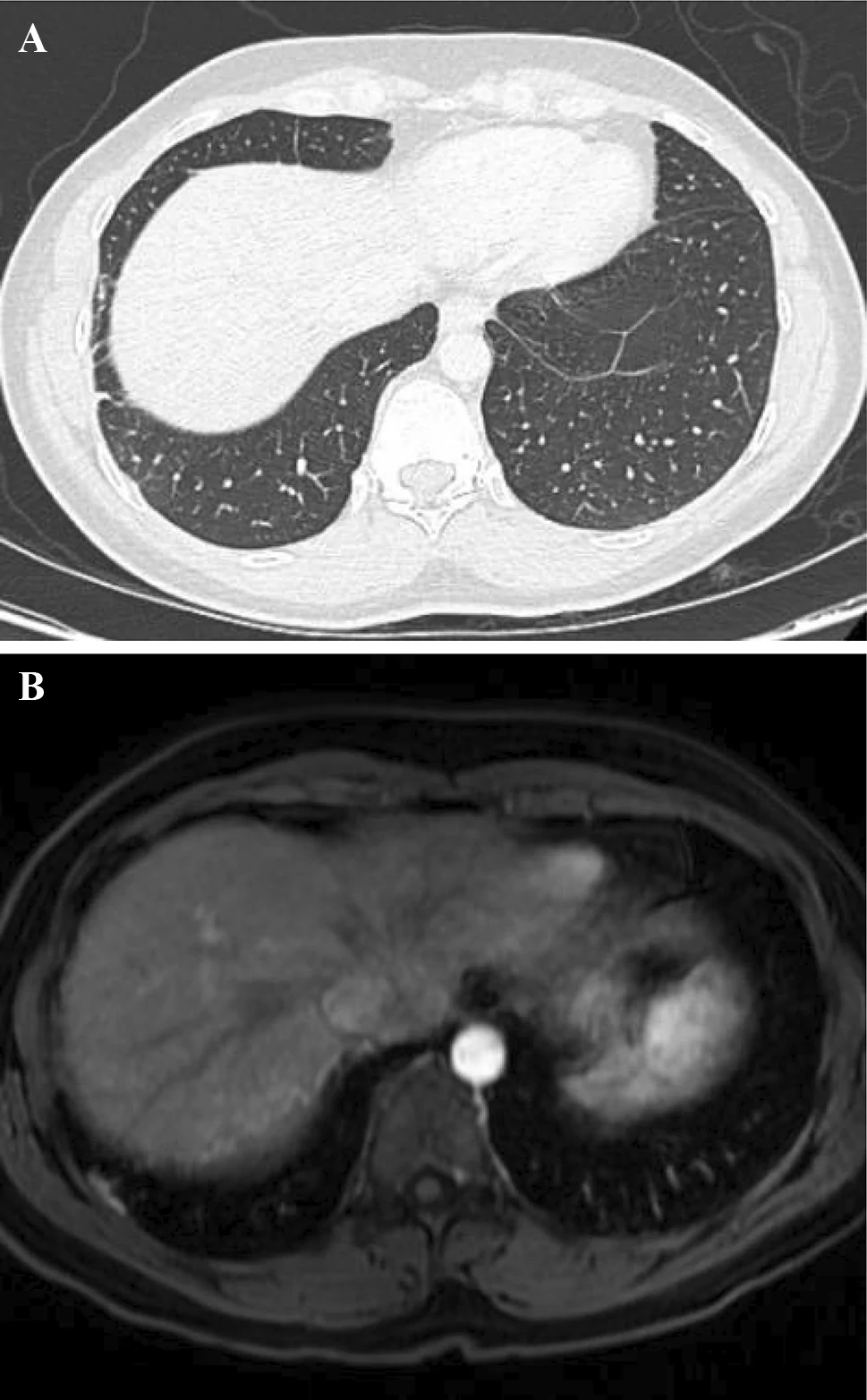
Computed tomography of chest [**A**] and magnetic resonance imaging of liver [**B**] with right pleural deposits and adhesions with atelectasis of the right lower lobe, possibly indicative of endometriosis and scarring on the diaphragm and/or the liver capsule, respectively

Computed Tomography (CT) chest and magnetic resonance imaging (MRI) liver obtained demonstrated right pleural deposits, right lower lobe (RLL) atelectasis (Fig. 1A), and suspected endometriosis involving the diaphragm and liver capsule (Fig. 1B). Multidisciplinary review with obstetrics and gynecology and thoracic surgery recommended operative intervention given persistent RUQ pain.

### Treatment pathway

**Fig. 2:**
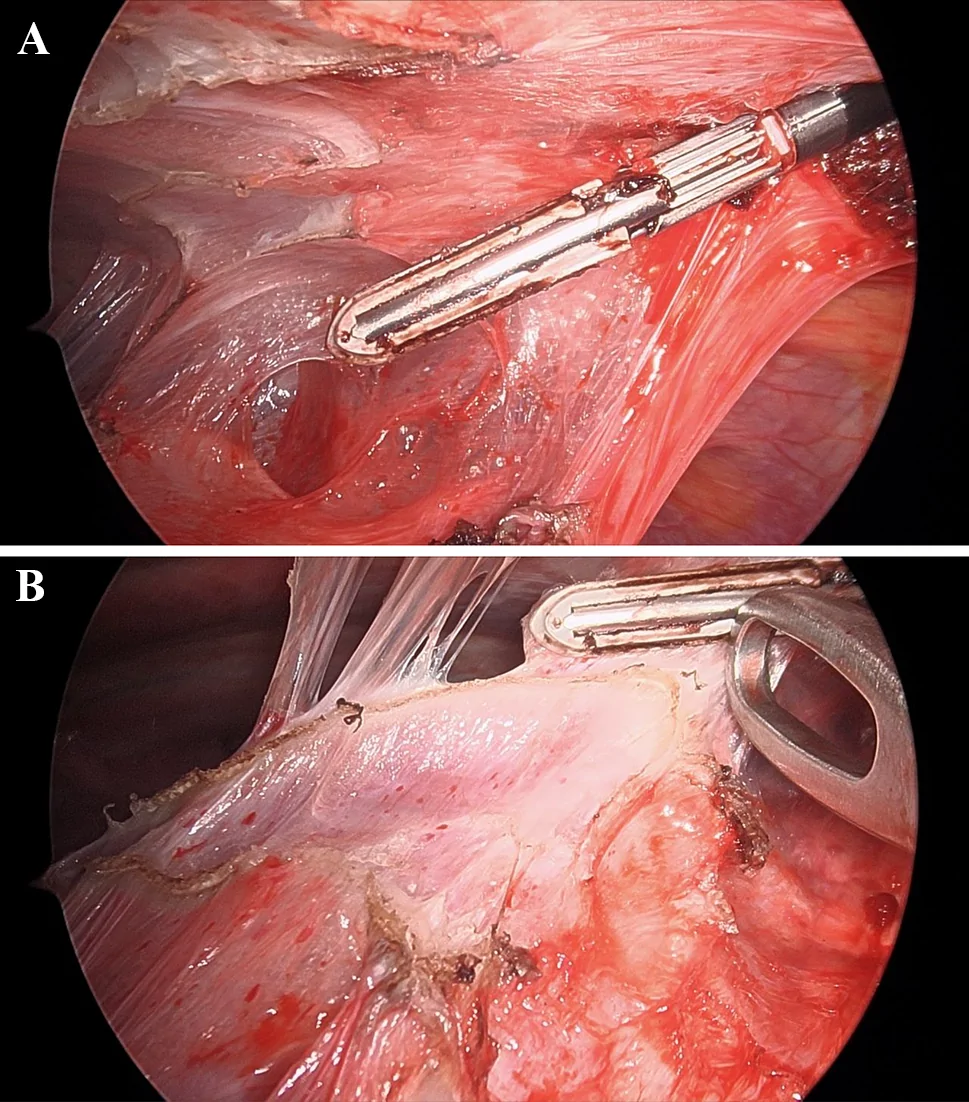
Right VATS exploration with dense adhesions of right lower lobe to diaphragm [**A**] with some nodularity, scar tissue, and chocolate colored deposits [**B**]

The patient was taken to the operating room. Initial bronchoscopy was normal and negative for masses. Right VATS revealed dense adhesions between the RLL and diaphragm (Fig. 2A) with nodularity, scarring, and chocolate-colored deposits (Fig. 2B) that were grossly characteristic of endometriosis based on size, texture, and color. Given the involvement of the parietal pleura, they were believed to be the source of the patient’s pain. A RLL wedge resection and full-thickness diaphragm resection was then performed. The diaphragm resection was performed using a blunt-tip ligasure, and the small defect was re-sutured under minimal tension with a 2 − 0 vicryl figure-of-eight suture. Adhesions were completely lysed and resected, and the sample was sent as RLL wedge and right diaphragm resection. VATS was followed by laparoscopic excision of pelvic brim lesions and lysis of adhesions along the abdominal wall and liver, without evidence of endometriosis on liver.

### Outcomes

Pathologic evaluation confirmed endometriosis in both thoracic and pelvic specimens. The patient recovered uneventfully and reported sustained resolution of symptoms at 1-, 4-, and 12-month follow-ups, and she remains symptom-free on longer-term follow-up after surgical intervention (December 2023).

## Discussion and Conclusions

Although thoracic endometriosis has been described in association with pleural and diaphragmatic disease, this case highlights under recognized features that expand clinical and surgical understanding of this condition. Prior reports describe presentations with catamenial pneumothorax or hemothorax and emphasize pleural or diaphragmatic involvement as the dominant pathology [3, 5, 9]. In contrast, this patient presented with a prolonged history of isolated cyclical RUQ pain without pulmonary manifestations, which contributed to a significant delay in diagnosis. Her symptoms were cyclical rather than classically catamenial and showed only partial or transient improvement with both non-suppressive and suppressive hormonal therapies, further contributing to diagnostic complexity. Additionally, this case demonstrates not only diaphragmatic, but also pathologically confirmed lung parenchymal involvement in the absence of hemoptysis or pneumothorax. While parenchymal thoracic endometriosis has been described, it is relatively uncommon and most frequently presents with catamenial hemoptysis [3, 5]. Absence of hallmark features in this patient highlights the heterogeneity of disease presentation and underscores the need to broaden clinical suspicion when evaluating reproductive-age women with cyclical thoracoabdominal pain. An important limitation is that histopathologic results from the prior appendectomy specimen were not available for review, which limits our ability to determine whether appendiceal endometriosis was present and whether it may have represented an earlier site of disease or a possible source of dissemination.

The patient’s persistent symptoms despite multiple forms of hormonal therapy supported the decision to pursue surgical management. VATS is the cornerstone of diagnosis and treatment and enables direct visualization and resection of pleural, parenchymal, and diaphragmatic lesions while minimizing morbidity compared with thoracotomy [7]. Laparoscopic evaluation of the abdomen and pelvis is crucial, as concurrent pelvic endometriosis occurs in 50–80% of cases; thus, simultaneous assessment is recommended and often pursued.

For diaphragmatic disease, techniques include Argon beam coagulation/diathermocoagulation, peritoneal stripping for plaques, and nodulectomy or full-thickness resection based on size, depth, and location of the lesion, while parenchymal disease is treated with resection. Given the dense adhesions and diaphragmatic lesions, adhesiolysis and wedge resection were performed as endometrial implants were localized to the RLL. All visual lesions were resected and ultimately confirmed pathologically. Full-thickness resection of the diaphragmatic lesions was performed, and the defect was closed primarily with 2 − 0 Vicryl figure-of-eight sutures, ensuring a tension-free repair. No obvious diaphragmatic fenestrations were observed, which are believed to be a key mechanism for the development of thoracic endometriosis [5]. In a surgical series of catamenial hemothorax, diaphragmatic fenestrations were identified in most patients and were managed with resection or synthetic patch repair in combination with pleural symphysis and postoperative hormonal therapy [10]. In this case, full-thickness resection was performed to mitigate known concerns about diaphragmatic fenestrations and to minimize the risk of recurrence [2]. The remaining defect was small enough to be closed primarily, and attention was paid to ensure the repair was tension-free to minimize the risk of future complications such as diaphragmatic hernia [11].

Complete excision of all visible endometrial implants is associated with long-term symptom control, while incomplete resection and lack of hormonal suppression may contribute to recurrence. Reported recurrence rates vary from 5 to 30%, underscoring the need for continued postoperative surveillance and consideration of adjunct hormonal therapy [8, 9]. Pleurodesis is often considered to obliterate the pleural space and reduce the risk of recurrence or future spontaneous pneumothorax by 20–25% [12]. In this case, no pleurodesis was performed since the patient had never experienced any prior bouts of pneumothoraces and/or pleural effusions. Additionally, avoidance of a potentially more invasive, chronic pain-inducing procedure would provide little benefit with increased risk for worsened pain.

A multidisciplinary approach to this case was critical given the presence of endometrial lesions involving the pelvic brim, diaphragm, and lung parenchyma. The involvement of obstetrics and gynecology, and thoracic surgery enabled single-stage resection of all visible lesions across both the thoracic and abdominopelvic compartments. This reduces the need for multiple procedures and minimizes persistent symptoms from untreated disease [8]. Intraoperatively, a multidisciplinary approach improved diagnostic accuracy in assessing disease burden across compartments.

Alternative management with hormonal therapy is well established for less severe cases of endometriosis. However, this patient had persistent and progressive symptoms despite prior hormonal therapy. Medical therapy may also fail to completely address intrinsic pathology, such as adhesions and parenchymal involvement [5]. As a result, surgical intervention was necessary to achieve the desired symptom relief and tissue diagnosis.

This case highlights the importance of a high index of suspicion in women presenting with cyclical RUQ pain and the value of a multidisciplinary, minimally invasive approach for definitive diagnosis and surgical management of thoracic and abdominopelvic endometriotic lesions for durable symptom resolution [8, 9]. In this patient, integration of thoracic surgery and obstetrics and gynecology in a single minimally invasive operation optimized diagnostic accuracy and treatment, with sustained symptom-free follow-up after surgery in late 2023.

## Data Availability

No datasets were generated or analysed during the current study.

## Abbreviations

CT: Computed tomography
MRI: Magnetic resonance imaging
RLL: Right lower lobe
RUQ: Right upper quadrant
VATS: Video-assisted thoracoscopic surgery
